# Response to treatment with intra-articular triamcinolone hexacetonide and triamcinolone acetonide in oligo articular juvenile idiopathic arthritis

**DOI:** 10.1186/s12969-021-00520-6

**Published:** 2021-03-20

**Authors:** Rana Harhay, Wajiha Jeelani, Barbine Tchamba Agbor Agbor, Teresa Hennon, Brian H. Wrotniak, Rabheh Abdul-Aziz

**Affiliations:** 1 Department of Pediatrics, University at Buffalo, Oishei Children’s Hospital, Buffalo, NY USA; 2 Department of Pediatric Rheumatology, University at Buffalo, Oishei Children’s Hospital, Buffalo, NY USA; 3 Department of Microbiology and Immunology, Jacobs School of Medicine and Biomedical Sciences, Buffalo, NY USA

**Keywords:** Oligo-articular juvenile idiopathic arthritis (Oligo JIA), Intra-articular corticosteroid (IAC), Triamcinolone hexacetonide (TH), Triamcinolone acetonide (TA)

## Abstract

**Background:**

Oligo-articular juvenile idiopathic arthritis (Oligo JIA) is the most common subtype of juvenile idiopathic arthritis. Intra-articular corticosteroid (IAC) injection is a mainstay treatment of oligo JIA providing pain relief, improving mobility and preventing further joint destruction in the majority of patients. In 2015, production of triamcinolone hexacetonide (TH) an intra-articular corticosteroid was discontinued in the United States leading to use of triamcinolone acetonide (TA) as an alternative. In this study, we compared response to treatment in children with oligo JIA who underwent therapy with intra-articular TA and TH injection.

**Methods:**

Our study is a retrospective chart review of children with oligo JIA who were treated with IAC injections with TH between January 2012 and June 2015 and TA between J uly 2015 and December 2018. The two groups were followed at John R. Oishei Children’s Hospital of Buffalo and were evaluated for response to treatment, side effects and predictors of response including duration of disease before treatment, erythrocyte sedimentation rate (ESR), and c-reactive protein (CRP). Response to treatment was defined as at least 6 months follow up without evidence of active arthritis in injected joints. Patients were considered to be non-responders if they continued to show active arthritis during their first follow up after joint injection. The primary objective was to evaluate whether there was a significant difference in rate of response between TH and TA.

**Results:**

Forty-nine patients, 38 female and 11 male with oligo JIA were included in the study. The average age was 6.7 years. A total of 111 joints were injected includin g 78 knees, 13 ankles, 9 wrists, 4 hips, 4 elbows, 2 TMJ and one subtalar joint. In the TA group, 49% (29/59) did not show response to injection compared to 27% (14/52) in the TH group. After 6 months, response rates were better for individuals injected with TH compared to TA (73% vs. 51%). In general, response to intra-articular TH was superior to TA with *P* = .016 using chi-square test of independence. This difference in outcome was not influenced by other variables such as duration of illness before treatment (*P* value 0.784) or elevated ESR and CRP. No difference in side effects between the two groups were noted.

**Conclusion:**

Our results in conjunction with prior published data suggests that TH intra-articular joint injection in oligo JIA is superior to TA, although future controlled trials are necessary for confirmation. An effective, long lasting treatment can have a great impact on the outcome of these children.

## Background

As defined by the International League of Associations for Rheumatology (ILAR), juvenile idiopathic arthritis (JIA) is arthritis that occurs before the age of sixteen and lasts for at least 6 weeks [1]. Oligo JIA is the most common subgroup of JIA, accounting for at least 40% of all cases of the disease [2]. Oligo JIA is an insidious disease with asymmetric involvement of 1 to 4 joints, most commonly in the lower limbs. Symptoms can persist into adulthood leading to permanent damage of joints, contractures, and physical disability [3]. In 2011, guidelines were published for the management of oligo JIA [4]. The initial treatment consists of nonsteroidal anti- inflammatory drugs (NSAIDS) for approximately 8 weeks. Subsequently, if inflammation and pain continues or worsens, IACs, preferably triamcinolone hexacetonide (TH) is warranted [4]. Historically, the intraarticular corticosteroid triamcinolone hexacetonide has provided longer lasting control of arthritis in patients with oligo JIA than its counterpart, triamcinolone acetonide (TA) [5, 6]. Since 2015, TH has not been available for use in the United States, and TA has been used instead as the alternative treatment [7]. In this study, we aim to report the response and compare the efficacy of IAC injection with TH and TA in patients with Oligo JIA.

## Methods

University at Buffalo Institutional Review Board approved this retrospective chart review study. Patients who followed at the Rheumatology Department at John R. Oishei Children’s Hospital of Buffalo were identified by the diagnostic codes of oligo JIA (ICD- 10 code: M08.40). The data for the retrospective chart review was collected using the Powerchart Electronic Medical Record.

### Subjects

The population for this study were children with a diagnosis of oligo articular JIA according to the ILAR classification criteria (2nd revision) [1] who received IAC injection with either TH between January 2012 and June 2015 or TA between July 2015 and December 2018. As TH was no longer available after 2015, TA was used as an alternative therapy.

Inclusion criteria included: diagnosis of oligo JIA, age less than 16 years, treatment with IAC joint injection with either TH or TA, and follow up within at least 6 months after the IAC joint injection. Exclusion criteria included use of disease-modifying anti rheumatic drugs (DMARDs) and/or failure to follow up within a 6-months period. No. patients who met these criteria were excluded from the study.

### Clinical characteristics

The following data were recorded: age, sex, clinical presentation at onset of disease, erythrocyte sedimentation rate (ESR), c-reactive protein (CRP), antinuclear antibody (ANA), rheumatic factor (RF), human leukocyte antigen B27 (HLA-B27), anti-cyclic citrullinated peptide (anti*-*CCP), the type of IAC used, disease duration before presentation, time of follow-up, and the response to treatment.

### Procedure

Two rheumatologists in the rheumatology department at Oishei Children’s Hospital performed most IAC injections. Six joints were injected by the interventional radiology department at Oishei Children’s Hospital and one joint was injected by a sports medicine provider not affiliated with our institution; the former due to the need for ultrasound guidance, and the latter due to the patient being out of state at the time of a flare up. The doses used for TH were 1 mg/kg for large joints (knees and hips) and 0.5 mg/kg for smaller joints (ankles, wrists, and elbows) [8]. For TA, higher dose was used, ranging from 1.2–1.7 mg/kg for large joints. All patients had a detailed musculoskeletal exam documented at baseline prior to IAC joint injection, and at any follow up visit after the IAC joint injection.

### Outcome

The presence of active arthritis was determined by the presence of pain with motion, swelling, limitation in passive range of motion, or warmth. A responder is defined as a patient with at least 6 months of sustained absence of active arthritis after IAC injection. A non- responder is defined as a patient with active arthritis during their first follow up after IAC injection. The initial follow up appointment after IAC injection ranged from one to 4 months. We recorded the duration of response to treatment in the responder group.

### Data analysis

Descriptive characteristics were computed to characterize the study sample. Gender was reported as a proportion in percentage, and continuous level variables (e.g., age at onset of disease) as means and range. A chi square tests of independence was used to compare the difference in response rate between TH and TA joint injections. A chi square test also was used to compare outcome of only knee joint injection with TH and TA. Likewise, separate Fisher’s exact tests were used to assess for differences in response rates between patients with elevated and normal CRP levels and for differences in response rates between patients with elevated and normal ESR levels. An independent t-test was used to investigate whether the duration of response to IAC joint injections differed for TH versus TA. Analyses were conducted assuming two-tailed hypotheses, an alpha of 0.05, and using SYSTAT 13 (SYSTAT Software, 2004).

## Results

We identified 65 patients with oligo JIA. Sixteen patients did not receive IAC joint injection due to family desire to try less invasive treatments or loss of follow up. The remaining 49 patients underwent IAC joint injections. The demographic data is summarized in Table 1. Other than four patients who were diagnosed after 24, 36, 48.and 72 months of symptom onset, patients were diagnosed within 2 months of disease onset.

**Table 1:** Demographic data and type of injected joint

**Number of Patients**		49
**Female**		38/49 (78%)
**Male**		11/49 (22%)
**Mean age at onset of disease (years)**		6.7 (Range 1–15)
**Mean disease duration (months)**		7 (Range 1–72)
**Injected Joints**	Number	Percent
**Knee**	78	70
**Ankle**	13	12
**Elbow**	4	3.6
**Hip**	4	3.6
**Wrist**	9	8.1
**Temporomandibular**	2	1.8
**Subtalar**	1	0.9
**Total**	111	

One hundred nineteen IAC joint injections were performed on 49 patients. Of the 119 IAC joint injections performed, 8 were excluded. Two injected joints were excluded due to lack of follow up after injection to assess for response, 5 due to disease modifying agents (DMARDS) use at any time after IAC joint injection, and 1 for using methylprednisolone IAC joint injection after initial joint injection with TH or TA. The remaining 111 joints that were injected included 78 knees (70.3%), 13 ankles (11.7%), 9 wrists (8.1%), 4 hips (3.6%), 4 elbows (3.6%), 2 TMJs (1.8%) and one subtalar. joint (0.9%) (Table 1).

Overall, 61% (68/111) of joints responded to either TH or TA IAC injection. A greater percentage of joints treated with TH sustained response 73% (38 of 52) compared with 51% (30 of 59) of joints treated with TA (*p* = 0.016). At the initial follow up appointment between 1 and 4 months after injection, 27% (14/52) joints in the TH group showed active arthritis compared to 49% (29/59) joints in the TA group. Within the first year after receiving a joint injection with TH or TA, 11.9% (7/59) of joints injected with TA had to be reinjected compared to only 3.8% (2/52) in the TH group. To avoid bias based on the type of injected joint we compared the outcome of only knee IAC injection. We have 78 knee IAC injection. A greater percentage of knees treated with TH sustained response 84% (31 of 37) compared with 49% (20 of 41) of knees treated with TA (*p* = 0.001).

Laboratory characteristics showed positive ANA in 23 patients and none with positive RF or positive anti- CCP. There is a 12 years old girl and a 5 years old boy with positive HLA- B27. Of the patients with elevated CRP, 83% (5/6) responded to IAC joint injection compared to 65% (15/23) of patients with normal CRP. Of the patients with elevated ESR, 88% (14/16) responded to joint injection while 60% (14/23) of patients with normal ESR did not respond. Despite the magnitude of the difference in response between elevated and normal CRP and ESR levels, the results did not reach statistical significance due to the relatively small sample sizes.

When examining the duration of response after IAC joint injection, we found that the duration of response for TH was significantly longer (2.66 years) than the response duration for TA which was 1.70 years (*p* = 0.026) (Table 2).

**Table 2:** Duration of response to IAC joint injection

Duration of Response	Response TH (joints number)	Response to TA (joints number)
**6 month**	8	4
**1 year**	10	11
2 **year**	3	9
**3 year**	2	4
**4 year**	6	2
**5 year**	6	0
**7 year**	3	0

Total number of responded joint 68/111 (61%)

Response to TH 38/52 (73%).

Response to TA 30/59 (51%)

Intra-articular steroid treatment was generally safe with only two patients exhibiting subcutaneous atrophy at the injection site, one from the TH group and one from TA. No complications such as joint infections, bleeding or chemical synovitis were reported (Table 3).

**Table 3:** TH and TA safety

Complication	Number of joints
Subcutaneous atrophy	1 joint injection with TH and 1 with TA
Infection	0
Bleeding	0

## Discussion

In children with JIA, IAC can provide pain relief, improve mobility, and delay or even prevent irreversible joint destruction [9–13]. TH and TA are among the most common medications used for IAC in JIA patients. The efficacy of TH has been studied and reported in the literature with rates of remission at 6 months ranging from 63.3 to 82% [9, 14, 15]. Sample sizes were as large as 794 joint injections in some studies [16]. The efficacy of TA is reported with 6 month response rates varying from 53.4 to 100% [17–19]. It is notable that studies of TA had small sample sizes ranging from 11 to 26 patients [17–19].

TH has not been available in United States since 2015 and from our knowledge there are no studies showing response to use of TA over the last 4 years. Before 2015, there were a limited number of studies that compared TH and TA efficacy in oligo JIA [5, 6, 16, 20]. Most of these studies found TH to be superior to TA at both the same and increased doses with different percentages of responses [5, 6, 16, 20].

The results of our study are consistent with previous publications demonstrating the superior efficacy of TH over TA. In our study, after 6 months of IAC injection 73% of injected joints with TH group had sustained response compared to 50.8% in TA group. Twenty seven percent of joints injected with TH showed active arthritis 1–4 months after treatment compared to 49% of joints in the TA group showing no response. TH showed more efficacy despite using higher dose of TA in some of our patients.

Our study is unique from past research in that it examined a longer duration of follow up. Most of the previous studies showed response only at 6 months, 12 months and maximum 24 months [5, 6]. We have patients showing response for 3–7 years after a single joint injection particularly in the TH group. The duration of response for TH was 2.658 years and for TA was 1.70 years (*p* = 0.026) showing superior effect of TH for a longer duration.

We analyzed different variables that may affect the efficacy of IAC joint injection including inflammatory markers and duration of disease before treatment. Results did not show a statistically significant difference in the rate of sustained response for 6 months after treatment with TH or TA between patients with elevated or normal CRP and ESR, though we cannot rule out type two error due to the small sample size.

Literature showed mixed results, Ravelli et al. found children with high ESR were more likely to have sustained response [21] while Hertzberger-ten Cate et al. found no correlation with ESR [22]. Lanni et al. showed risk of synovitis flare was higher in patients who had elevated CRP which is not consistent with our study [23].

We found no relationship between response and duration of disease before treatment (*p* = 0.784). In the study by Allen et al. they found favorable outcome was correlated with short disease duration [15].

In terms of adverse side effects to treatment with TH or TA in our study, the treatments were well tolerated.

## Conclusion

The results of our study are comparable to the few studies published that indicate the superiority of TH compared to TA in the sustained treatment of oligo JIA. Many factors may affect the efficacy of this treatment modality, however the majority of evidence on this topic is largely inconclusive, anecdotal, or conflicting, with mixed results pertaining to disease duration, ESR, and CRP. One limitation of our study is the retrospective nature and small sample size. In addition, we have 2 patients with positive HLA 27 and 2 patients with TMJ involvement that is not typical for oligo JIA. Larger prospective studies analyzing the response to TA and predictors of response including the medication dose should be considered to confirm our findings.

## Data Availability

Upon request.

## Abbreviations

Oligo JIA: Oligo-articular juvenile idiopathic arthritis
IAC: Intra-articular corticosteroid
TH: Triamcinilone hexacetonide
TA: Triamcinilone acetonide
ESR: Erythrocyte Sediminatation Rate
ILAR: International League of Associations for Rheumatology
CRP: C-reactive protein
DMARDs: Disease modifying anti-rheumatic Drugs
